# An Iterative Algorithm for the Reflexive Solution of the General Coupled Matrix Equations

**DOI:** 10.1155/2013/952974

**Published:** 2013-11-11

**Authors:** Zhongli Zhou, Guangxin Huang

**Affiliations:** Geomathematics Key Laboratory of Sichuan Province, College of Management Science, Chengdu University of Technology, Chengdu 610059, China

## Abstract

The general coupled matrix equations (including the generalized coupled Sylvester matrix equations as special cases) have numerous applications in control and system theory. In this paper, an iterative algorithm is constructed to solve the general coupled matrix equations over reflexive matrix solution. When the general coupled matrix equations are consistent over reflexive matrices, the reflexive solution can be determined automatically by the iterative algorithm within finite iterative steps in the absence of round-off errors. The least Frobenius norm reflexive solution of the general coupled matrix equations can be derived when an appropriate initial matrix is chosen. Furthermore, the unique optimal approximation reflexive solution to a given matrix group in Frobenius norm can be derived by finding the least-norm reflexive solution of the corresponding general coupled matrix equations. A numerical example is given to illustrate the effectiveness of the proposed iterative algorithm.

## 1. Introduction

Let *P* ∈ *ℛ* ^*n*×*n*^ be a generalized reflection matrix; that is, *P* ^*T*^ = *P* and *P* ^2^ = *I*. A matrix *A* ∈ *ℛ* ^*n*×*n*^ is called reflexive with respect to the matrix *P* if *PAP* = *A*. The set of all *n*-by-*n* reflexive matrices with respect to the generalized reflection matrix *P* is denoted by *ℛ*_*r*_^*n*×*n*^(*P*). Let *ℛ* ^*m*×*n*^ denote the set of all *m* × *n* real matrices. We denote by the superscript *T* the transpose of a matrix. In matrix space *ℛ* ^*m*×*n*^, define inner product as; 〈*A*, *B*〉 = tr⁡(*B* ^*T*^ *A*) for all *A*, *B* ∈ *ℛ* ^*m*×*n*^; ||*A*||_*F*_ represents the Frobenius norm of *A*. *ℛ*(*A*) represents the column space of *A*. *vec*⁡(·) represents the vector operator; that is, *vec*⁡(*A*) = (*a*_1_^*T*^, *a*_2_^*T*^,…, *a*_*n*_^*T*^)^*T*^ ∈ *ℛ* ^*mn*^ for the matrix *A* = (*a*_1_, *a*_2_,…, *a*_*n*_) ∈ *ℛ* ^*m*×*n*^, *a*_*i*_ ∈ *R* ^*m*^, *i* = 1,2,…, *n*. *A* ⊗ *B* stands for the Kronecker product of matrices *A* and *B*. 

In this paper, we will consider the following two problems.

**Problem 1**

Let *P*_*j*_ ∈ *ℛ* ^*n*_*j*_×*n*_*j*_^ be generalized reflection matrices. For given matrices *A*_*ij*_ ∈ *ℛ* ^*r*_*i*_×*n*_*j*_^, *B*_*ij*_ ∈ *ℛ* ^*n*_*j*_×*s*_*i*_^, and *M*_*i*_ ∈ *ℛ* ^*r*_*i*_×*s*_*i*_^, find reflexive matrix solution group (*X*_1_, *X*_2_,…, *X*_*q*_) with *X*_*j*_ ∈ *ℛ*_*r*_^*n*_*j*_×*n*_*j*_^(*P*_*j*_) such that

(1) $$\begin{array}{c} \sum_{j=1}^{q}A_{ij}X_{j}B_{ij}=M_{i},\;i=1,2,\ldots ,p. \end{array}$$

**Problem 2**

When Problem 1 is consistent, let *S*_*E*_ denote the set of the reflexive solution group of Problem 1; that is,

(2) SE={(X1,X2,…,Xq) ∣ ∑j=1qAijXjBij=Mi,   i=1,2,…,p,  Xj∈ℛrnj×nj(Pj)}.

For a given reflexive matrix group

(3) (X10,X20,…,Xq0)∈ℛrn1×n1(P1)  ×ℛrn2×n2(P2)×⋯×ℛrnq×nq(Pq),

Find $({\hat{X}}_{1},{\hat{X}}_{2},\ldots ,{\hat{X}}_{q})\in S_{E}$ such that

(4) ∑j=1q||X^j−Xj0||2  =min⁡(X1,X2,…,Xq)∈SE{∑j=1q||Xj−Xj0||2}.

The general coupled matrix equations (1) (including the generalized coupled Sylvester matrix equations as special cases) may arise in many areas of control and system theory.

 Many theoretical and numerical results on (1) and some of its special cases have been obtained. Least-squares-based iterative algorithms are very important in system identification, parameter estimation, and signal processing, including the recursive least squares (RLS) and iterative least squares (ILS) methods for solving the solutions of some matrix equations, for example, the Lyapunov matrix equation, Sylvester matrix equations, and coupled matrix equations as well. For example, novel gradient-based iterative (GI) method [1–5] and least-squares-based iterative methods [3, 4, 6] with highly computational efficiencies for solving (coupled) matrix equations are presented and have good stability performances, based on the hierarchical identification principle, which regards the unknown matrix as the system parameter matrix to be identified. Ding and Chen [1] presented the gradient-based iterative algorithms by applying the gradient search principle and the hierarchical identification principle for (1) with *q* = *p*. Wu et al. [7, 8] gave the finite iterative solutions to coupled Sylvester-conjugate matrix equations. Wu et al. [9] gave the finite iterative solutions to a class of complex matrix equations with conjugate and transpose of the unknowns. Jonsson and Kågström [10, 11] proposed recursive block algorithms for solving the coupled Sylvester matrix equations and the generalized Sylvester and Lyapunov Matrix equations. By extending the idea of conjugate gradient method, Dehghan and Hajarian [12] constructed an iterative algorithm to solve (1) with *q* = *p* over generalized bisymmetric matrices. Very recently, Huang et al. [13] presented a finite iterative algorithms for the one-sided and generalized coupled Sylvester matrix equations over generalized reflexive solutions. Yin et al. [14] presented a finite iterative algorithms for the two-sided and generalized coupled Sylvester matrix equations over reflexive solutions. For more results, we refer to [15–28]. However, to our knowledge, the reflexive solution to the general coupled matrix equations (1) and the optimal approximation reflexive solution have not been derived. In this paper, we will consider the reflexive solution of (1) and the optimal approximation reflexive solution. 

 This paper is organized as follows. In Section 2, we will solve Problem 1 by constructing an iterative algorithm. The convergence of the proposed algorithm is proved. For any arbitrary initial matrix group, we can obtain a reflexive solution group of Problem 1 within finite iteration steps in the absence of round-off errors. Furthermore, for a special initial matrix group, we can obtain the least Frobenius norm solution of Problem 1. Then in Section 3, we give the optimal approximate solution group of Problem 2 by finding the least Frobenius norm reflexive solution group of the corresponding general coupled matrix equations. In Section 4, a numerical example is given to illustrate the effectiveness of our method. At last, some conclusions are drawn in Section 5.

## 2. An Iterative Algorithm for Solving Problem 1

In this section, we will first introduce an iterative algorithm to solve Problem 1 then prove its convergence. We will also give the least-norm reflexive solution of Problem 1 when an appropriate initial iterative matrix group is chosen.

**Algorithm 3**

*Step  1*. Input matrices *A*_*ij*_ ∈ *ℛ* ^*r*_*i*_×*n*_*j*_^, *B*_*ij*_ ∈ *ℛ* ^*n*_*j*_×*s*_*i*_^, *M*_*i*_ ∈ *ℛ* ^*r*_*i*_×*s*_*i*_^, and generalized reflection matrices *P*_*j*_ ∈ *ℛ* ^*n*_*j*_×*n*_*j*_^, *i* = 1,…, *p*, *j* = 1,…, *q*.

*Step  2*. Choose an arbitrary matrix group

(5) (X1(1),X2(1),…,Xq(1))∈ℛrn1×n1(P1)  ×ℛrn2×n2(P2)×⋯×ℛrnq×nq(Pq).

Compute

(6) R(1)=diag⁡(M1−∑l=1qA1lXl(1)B1l,M2     −∑l=1qA2lXl(1)B2l,…,Mp−∑l=1qAplXl(1)Bpl),Sj(1)=12[∑i=1pAijT(Mi−∑l=1qAilXl(1)Bil)BijT     +∑i=1pPjAijT(Mi−∑l=1qAilXl(1)Bil)BijTPj],                   k:=1.

*Step  3*. If *R*(*k*) = 0, then stop and (*X*_1_(*k*), *X*_2_(*k*),…, *X*_*q*_(*k*)) is the solution group of (1); elseif *R*(*k*) ≠ 0, but *S*_*j*_(*k*) = 0, *j* = 1,…, *q*, then stop and (1) are not consistent over reflexive matrix group; else *k* : = *k* + 1.

*Step  4*. Compute

(7) Xj(k)=Xj(k−1)+||R(k−1)||F2∑l=1q||Sl(k−1)||F2    ×Sj(k−1), j=1,…,q,R(k)=diag⁡(M1−∑l=1qA1lXl(k)B1l,M2     −∑l=1qA2lXl(k)B2l,…,Mp−∑l=1qAplXl(k)Bpl)=R(k−1)−||R(k−1)||F2∑l=1q||Sl(k−1)||F2   ·diag⁡(∑l=1qA1lSl(k−1)B1l,∑l=1qA2lSl(k−1)B2l,…,      ×∑l=1qAplSl(k−1)Bpl),Sj(k)=12[∑i=1pAijT(Mi−∑l=1qAilXl(k)Bil)BijT     +∑i=1pPjAijT(Mi−∑l=1qAilXl(k)Bil)BijTPj]    +||R(k)||F2||R(k−1)||F2Sj(k−1).

*Step  5*. Go to Step  3.

Obviously, it can be seen that *X*_*j*_(*k*), *S*_*j*_(*k*) ∈ *R*_*r*_^*n*_*j*_×*n*_*j*_^(*P*_*j*_) for all *j* = 1,…, *q* and *k* = 1,2,….

**Lemma 4**

For the sequences {*R*(*k*)}, {*S*_*j*_(*k*)}  (*j* = 1,2,…, *q*) generated by Algorithm 3, and *m* ≥ 2, we have

(8) tr⁡((R(s))TR(t))=0,  ∑j=1qtr⁡((Sj(s))TSj(t))=0,             s,t=1,2,…,m,s≠t.

The proof of Lemma 4 is presented in the appendix.

**Lemma 5**

Suppose that (*X*_1_*, *X*_2_*,…, *X*_q_*) is an arbitrary reflexive solution group of Problem 1; then for any initial reflexive matrix group (*X*_1_(1), *X*_2_(1),…, *X*_*q*_(1)), one has

(9) $$\begin{array}{c} \sum_{j=1}^{q}\mathrm{tr}\left({\left(X_{j}^{*}-X_{j}\left(k\right)\right)}^{T}S_{j}\left(k\right)\right)={||R\left(k\right)||}_{F}^{2},\;k=1,2,\ldots , \end{array}$$

where the sequences {*X*_*j*_(*k*)}, {*S*_*j*_(*k*)}, and {*R*(*k*)} are generated by Algorithm 3.

The proof of Lemma 5 is presented in the appendix.

**Remark 6**

If there exists a positive number *k* such that *S*_*j*_(*k*) = 0, *j* = 1,2,…, *q* but *R*(*k*) ≠ 0, then, by Lemma 5, we get that (1) are not consistent over reflexive matrices.

**Theorem 7**

Suppose that Problem 1 is consistent; then for an arbitrary initial matrix group (*X*_1_, *X*_2_,…, *X*_*q*_) with *X*_*j*_ ∈ *ℛ*_*r*_^*n*_*j*_×*n*_*j*_^(*P*_*j*_), a reflexive solution group of Problem 1 can be obtained with finite iteration steps in the absence of round-off errors.

**Proof**

If *R*(*k*) ≠ 0, *k* = 1,2,…, *m* = ∑_*i*=1_^*p*^ *r*_*i*_ *s*_*i*_, then by Lemma 5 and Remark 6 we have *S*_*j*_(*k*) ≠ 0 for all *j* = 1,2,…, *q* and *k* = 1,2,…, *m*. Thus we can compute *R*(*m* + 1) and (*X*_1_(*m* + 1), *X*_2_(*m* + 1),…, *X*_*q*_(*m* + 1)) by Algorithm 3.

By Lemma 4, we have

(10) $$\begin{array}{c} \mathrm{tr}\left({\left(R\left(m+1\right)\right)}^{T}R\left(k\right)\right)=0,\;k=1,2,\ldots ,m,\\ \mathrm{tr}\left({\left(R\left(k\right)\right)}^{T}R\left(l\right)\right)=0,\;k,l=1,2,\ldots ,m,\;\;k\neq l. \end{array}$$

It can be seen that the set of *R*(1), *R*(2),…, *R*(*m*) is an orthogonal basis of the matrix subspace

(11) S={L ∣ L=diag⁡(L1,L2,…,Lp),   Li∈ℛri×si,  i=1,2…,p},

which implies that *R*(*m* + 1) = 0; that is, (*X*_1_(*m* + 1), *X*_2_(*m* + 1),…, *X*_*q*_(*m* + 1)) with *X*_*j*_(*m* + 1) ∈ *ℛ*_*r*_^*n*_*j*_×*n*_*j*_^(*P*_*j*_) is a reflexive solution group of Problem 1. This completes the proof.

To show the least Frobenius norm reflexive solution of Problem 1, we first introduce the following result.

**Lemma 8 (see [20, Lemma 2.4])**

Suppose that the consistent system of linear equation *Ax* = *b* has a solution x* ∈ *R*(*A* ^*T*^); then x* is a unique least Frobenius norm solution of the system of linear equation.

By Lemma 8, the following result can be obtained.

**Theorem 9**

Suppose that Problem 1 is consistent. If one chooses the initial iterative matrices *X*_*j*_(1) = ∑_*i*=1_^*p*^ *A*_*ij*_^*T*^ *K*_*i*_ *B*_*ij*_^*T*^ + ∑_*i*=1_^*p*^ *P*_*j*_ *A*_*ij*_^*T*^ *K*_*i*_ *B*_*ij*_^*T*^ *P*_*j*_, *j* = 1,2,…, *q*, where *K*_*i*_ ∈ *ℛ* ^*r*_*i*_×*s*_*i*_^, *i* = 1,2,…, *p* are arbitrary matrices, especially, *X*_*j*_(1) = 0 ∈ *ℛ* ^*n*_*j*_×*n*_*j*_^(*P*_*j*_), then the solution group (*X*_1_*, *X*_2_*,…, *X*_q_*) generated by Algorithm 3 is the unique least Frobenius norm reflexive solution group of Problem 1.

**Proof**

We know that the solvability of (1) over reflexive matrices is equivalent to the following matrix equations:

(12) ∑j=1qAijXjBij=Mi (i=1,2,…,p),∑j=1qAijPjXjPjBij=Mi (i=1,2,…,p).

Then the system of matrix equations (12) is equivalent to

(13) (B11T⊗A11⋯B1qT⊗A1q⋮⋯⋮Bp1T⊗Ap1⋯BpqT⊗ApqB11TP1⊗A11P1⋯B1qTPq⊗A1qPq⋮⋯⋮Bp1TP1⊗Ap1P1⋯BpqTPq⊗ApqPq)   ×(vec⁡(X1)⋮vec⁡(Xq))=(vec⁡(M1)⋮vec⁡(Mp)vec⁡(M1)⋮vec⁡(Mp)).

Let *X*_*j*_(1) = ∑_*i*=1_^*p*^ *A*_*ij*_^*T*^ *K*_*i*_ *B*_*ij*_^*T*^ + ∑_*i*=1_^*p*^ *P*_*j*_ *A*_*ij*_^*T*^ *K*_*i*_ *B*_*ij*_^*T*^ *P*_*j*_, *j* = 1,2,…, *q*, where *K*_*i*_ ∈ *ℛ* ^*r*_*i*_×*s*_*i*_^ are arbitrary matrices; then

(14) (vec⁡(X1(1))⋮vec⁡(Xq(1)))=(vec⁡(∑i=1pAi1TKiBi1T+∑i=1pP1Ai1TKiBi1TP1)⋮vec⁡(∑i=1pAiqTKiBiqT+∑i=1pPqAiqTKiBiqTPq))=(B11⊗A11T⋯Bp1⊗Ap1TP1B11⊗P1A11T⋯P1Bp1⊗P1Ap1T⋮⋯⋮⋯⋯⋮B1q⊗A1qT⋯Bpq⊗ApqTPqB1q⊗PqA1qT⋯PqBpq⊗PqApqT)(vec⁡(K1)⋮vec⁡(Kp)vec⁡(K1)⋮vec⁡(Kp))=(B11T⊗A11⋯B1qT⊗A1q⋮⋮⋮Bp1T⊗Ap1⋯BpqT⊗ApqB11TP1⊗A11P1⋯B1qTPq⊗A1qPq⋮⋮⋮Bp1TP1⊗Ap1P1⋯BpqTPq⊗ApqPq)T(vec⁡(K1)⋮vec⁡(Kp)vec⁡(K1)⋮vec⁡(Kp))∈R((B11T⊗A11⋯B1qT⊗A1q⋮⋮⋮Bp1T⊗Ap1⋯BpqT⊗ApqB11TP1⊗A11P1⋯B1qTPq⊗A1qPq⋮⋮⋮Bp1TP1⊗Ap1P1⋯BpqTPq⊗ApqPq)T).

Furthermore, we can see that all reflexive matrix solution groups (*X*_1_(*k*), *X*_2_(*k*),…, *X*_*q*_(*k*)) generated by Algorithm 3 satisfy

(15) (vec⁡(X1(1))⋮vec⁡(Xq(1))) ∈R((B11T⊗A11⋯B1qT⊗A1q⋮⋮⋮Bp1T⊗Ap1⋯BpqT⊗ApqB11TP1⊗A11P1⋯B1qTPq⊗A1qPq⋮⋮⋮Bp1TP1⊗Ap1P1⋯BpqTPq⊗ApqPq)T);

by Lemma 8 we know that (*X*_1_*, *X*_2_*,…, *X*_q_*) is the least Frobenius norm reflexive solution group of the system of linear equation (13). Since vector operator is isomorphic, (*X*_1_*, *X*_2_*,…, *X*_q_*) is the unique least Frobenius norm reflexive solution group of the system of matrix equations (12). Thus (*X*_1_*, *X*_2_*,…, *X*_q_*) is the unique least Frobenius norm reflexive solution group of Problem 1. This completes the proof.

## 3. The Solution of Problem 2

In this section, we will show that the reflexive solution group of Problem 2 to a given reflexive matrix group can be derived by finding the least Frobenius norm reflexive solution group of the corresponding general coupled matrix equations. 

 When Problem 1 is consistent, the set of the reflexive solution groups of Problem 1 denoted by *S*_*E*_ is not empty. For a given matrix pair (*X*_1_^0^, *X*_2_^0^,…, *X*_*q*_^0^) with *X*_*j*_^0^ ∈ *ℛ*_*r*_^*n*_*j*_×*n*_*j*_^(*P*_*j*_), *j* = 1,2,…, *q*, we have

(16) ∑j=1qAijXjBij=Mi⇔∑j=1qAij(Xj−Xj0)Bij=Mi−∑j=1qAijXj0Bij,   i=1,2,…,p.

Set ${\tilde{X}}_{j}=X_{j}-X_{j}^{0}$ and ${\tilde{M}}_{i}=M_{i}-\sum_{j=1}^{q}A_{ij}X_{j}^{0}B_{ij}$; then solving Problem 2 is equivalent to finding the least Frobenius norm reflexive solution group $\left({\tilde{X}}_{1}^{*},{\tilde{X}}_{2}^{*},\ldots ,{\tilde{X}}_{q}^{*}\right)$ of the corresponding general coupled matrix equations

(17) $$\begin{array}{c} \sum_{j=1}^{q}A_{ij}{\tilde{X}}_{j}B_{ij}={\tilde{M}}_{i},\;i=1,2,\ldots ,p. \end{array}$$

By using Algorithm 3, let initial iteration matrices

(18) X~j(1)=∑i=1pAijTKiBijT   +∑i=1pPjAijTKiBijTPj, j=1,2,…,q,

where *K*_*i*_ ∈ *ℛ* ^*r*_*i*_×*s*_*i*_^, *i* = 1,2,…, *p* are arbitrary matrices, especially, ${\tilde{X}}_{j}\left(1\right)=0\in {ℛ}^{n_{j}\times n_{j}}\left(P_{j}\right)$, *j* = 1,2,…, *q*; then we can get the the least Frobenius norm reflexive solution group $\left({\tilde{X}}_{1}^{*},{\tilde{X}}_{2}^{*},\ldots ,{\tilde{X}}_{q}^{*}\right)$ of (17). Thus the reflexive solution group of Problem 2 can be represented as

(19) $$\begin{array}{c} \left({\hat{X}}_{1},{\hat{X}}_{2},\ldots ,{\hat{X}}_{q}\right)=\left({\tilde{X}}_{1}^{*}+X_{1}^{0},{\tilde{X}}_{2}^{*}+X_{2}^{0},\ldots ,{\tilde{X}}_{q}^{*}+X_{q}^{0}\right). \end{array}$$

## 4. A Numerical Example

In this section, we will show a numerical example to illustrate our results. All the tests are performed by MATLAB 7.8.

**Example 10**

Consider the reflexive solution of the general coupled matrix equations

(20) A11X1B11+A12X2B12=M1,A21X1B21+A22X2B22=M2,

where

(21) A11=(13−57−92046−10−296−83622−3−55−22−1−1184−6−9−9),

(22) B11=(356748−54−15−23392−6−27−81),

(23) A12=(6−57−9246−119−123−81364−15−515−13−1129−6−9),

Let

(24) P1=(000100000100−1001000001000),  P2=(0010000−110000−100)

be the generalized reflection matrices.

We will find the reflexive solution of the the general coupled matrix equations (20) by using Algorithm 3. It can be verified that the matrix equations (20) are consistent over reflexive matrices and the solution is

(25) X1∗=(30−63−443−64−20240−23−46304−2643),X2∗=(−52−112−12−3−1−1−5−2−2−3−2−1).

Because of the influence of the error of calculation, the residual *R*(*k*) is usually unequal to zero in the process of the iteration, where *k* = 1,2,…. For any chosen positive number *ε*, however small enough, for example, *ε* = 1.0000*e* − 010, whenever ||*R*(*k*)|| < *ε*, stop the iteration; (*X*_1_(*k*), *X*_2_(*k*)) is regarded to be the reflexive solution of the matrix equations (20). Choose an initially iterative matrix group (*X*_1_(1), *X*_2_(1)), such as

(26) X1(1)=(0000000000000000000000000),  X2(1)=(0000000000000000);

by Algorithm 3, we have

(27) X1∗=X1(31) =(3.0000−0.0000−6.00003.0000−4.00004.00003.0000−6.00004.0000−2.00000.00002.00004.0000−0.0000−2.00003.0000−4.00006.00003.0000−0.00004.0000−2.00006.00004.00003.0000),X2∗=X2(31)=(−5.00002.0000−1.00001.00002.0000−1.00002.0000−3.0000−1.0000−1.0000−5.0000−2.0000−2.0000−3.0000−2.0000−1.0000),||R(31)||=3.1869e−011<ε.

**Figure 1:**
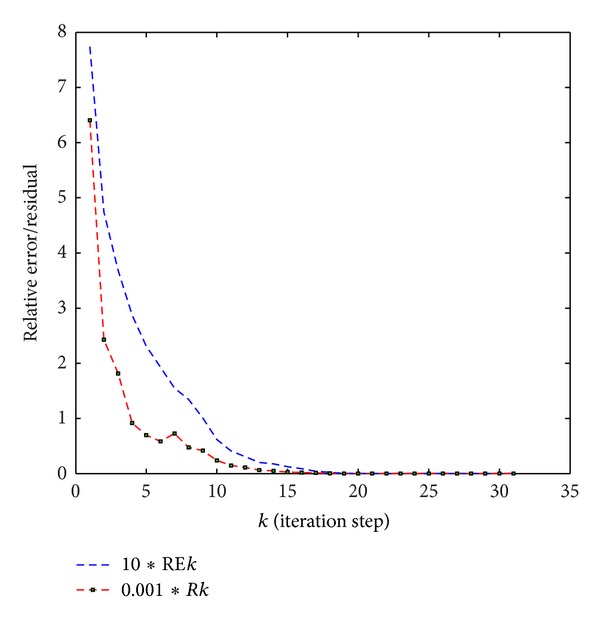
The relative error of the solutions and the residual for Example 10 with *X*_1_(1) = 0 and *X*_2_(1) = 0.

So we obtain the reflexive solution of the matrix equations (20). The relative error of the solution and the residual are shown in Figure 1, where the relative error *REk* = (||*X*_1_(*k*) − *X*_1_*|| + ||*X*_2_(*k*) − *X*_2_*||)/(||*X*_1_*|| + ||*X*_2_*||) and the residual *Rk* = ||*R*(*k*)||.

Let *S*_*E*_ denote the set of all reflexive solution group of the matrix equations (20). For two given reflexive matrices,

(28) X10=(23−533−133−525−22−5233523−52−3−13),X20=(−3−34201124−2−33−1201),

we will find $({\hat{X}}_{1},{\hat{X}}_{2})\in S_{E}$, such that

(29) ||X^1−X10||+||X^2−X20||  =min⁡(X1,X2)∈SE||X1−X10||+||X2−X10||;

that is, find the optimal approximate reflexive solution group to the given matrix group (*X*_1_^0^, *X*_2_^0^) in *S*_*E*_ in Frobenius norm.

Let ${\tilde{X}}_{1}=X_{1}-X_{1}^{0}$, ${\tilde{X}}_{2}=X_{2}-X_{2}^{0}$, ${\tilde{M}}_{1}=M_{1}-A_{11}X_{1}^{0}B_{11}-A_{12}X_{2}^{0}B_{12}$, ${\tilde{M}}_{2}=M_{2}-A_{21}X_{1}^{0}B_{21}-A_{22}X_{2}^{0}B_{22}$, by the method mentioned in Section 3, we can obtain the least-norm reflexive solution group $\left({\tilde{X}}_{1}^{*},{\tilde{X}}_{2}^{*}\right)$ of the matrix equations $A_{11}{\tilde{X}}_{1}B_{11}+A_{12}{\tilde{X}}_{2}B_{12}={\tilde{M}}_{1}$ and $A_{21}{\tilde{X}}_{1}B_{21}+A_{22}{\tilde{X}}_{2}B_{22}={\tilde{M}}_{2}$ by choosing the initially iterative matrices ${\tilde{X}}_{1}(1)=0$ and ${\tilde{X}}_{2}(1)=0$; then by Algorithm 3 we have that

(30) X~1∗=X~1(29) =(1.0000−3.0000−1.0000−0.0000−7.00005.00000.0000−9.00009.0000−4.0000−5.00004.00002.00005.0000−4.0000−0.0000−7.00001.00001.0000−3.00009.0000−4.00009.00005.0000−0.0000),X~2∗=X~2(29)=(−2.00005.0000−5.0000−1.00002.0000−2.00001.0000−5.0000−5.00001.0000−2.0000−5.0000−1.0000−5.0000−2.0000−2.0000),  ||R(30)||=3.6134e−011<ε=1.0000e−010,

and the optimal approximate reflexive solution to the matrix group (*X*_1_^0^, *X*_2_^0^) in Frobenius norm are

(31) X^1=X~1∗+X10 =(3.00000.0000−6.00003.0000−4.00004.00003.0000−6.00004.0000−2.00000.00002.00004.0000−0.0000−2.00003.0000−4.00006.00003.00000.00004.0000−2.00006.00004.00003.0000),X^2=X~2∗+X20=(−5.00002.0000−1.00001.00002.0000−1.00002.0000−3.0000−1.0000−1.0000−5.0000−2.0000−2.0000−3.0000−2.0000−1.0000).

**Figure 2:**
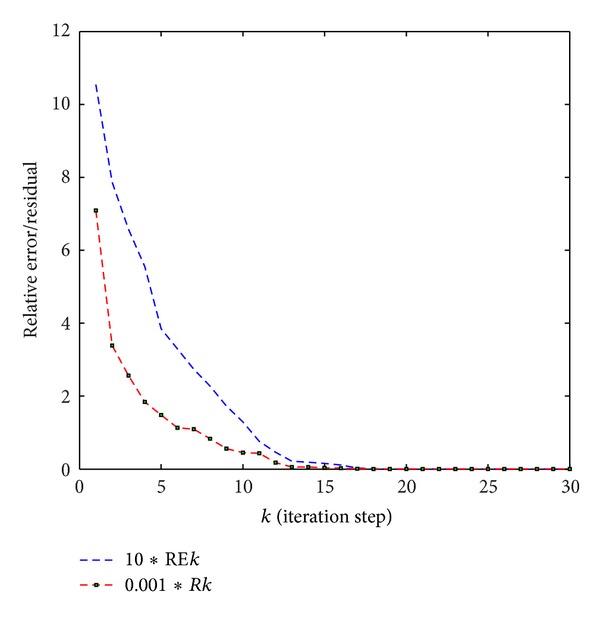
The relative error of the solutions and the residual for Example 10 with *X*_1_^0^ and *X*_2_^0^.

The relative error and the residual of the solution are shown in Figure 2, where the relative error $REk=\left(||{\tilde{X}}_{1}(k)+X_{1}^{0}-X_{1}^{*}||+||{\tilde{X}}_{2}(k)+X_{2}^{0}-X_{2}^{*}||\right)/\left(||X_{1}^{*}||+||X_{2}^{*}||\right)$ and the residual *Rk* = ||*R*(*k*)||.

## 5. Conclusions

 In this paper, an iterative algorithm is presented to solve the general coupled matrix equations ∑_*j*=1_^*q*^ *A*_*ij*_ *X*_*j*_ *B*_*ij*_ = *M*_*i*_  (*i* = 1,2,…, *p*) over reflexive matrices. When the general coupled matrix equations are consistent over reflexive matrices, for any initially reflexive matrix group, the reflexive solution group can be obtained by the iterative algorithm within finite iterative steps in the absence of round-off errors. When a special kind of initial iteration matrix group is given, the unique least-norm reflexive solution of the general coupled matrix equations can be derived. Furthermore, the optimal approximate reflexive solution of the general coupled matrix equations to a given reflexive matrix group can be derived by finding the least-norm reflexive solution of new corresponding general coupled matrix equations. Finally, a numerical example is given in Section 4 to illustrate that our iterative algorithm is quite effective.

## Appendices

### A. The Proof of Lemma 4

Since tr⁡((*R*(*s*))^*T*^ *R*(*t*)) = tr⁡((*R*(*t*))^*T*^ *R*(*s*)) and tr⁡((*S*_*j*_(*s*))^*T*^ *S*_*j*_(*t*)) = tr⁡((*S*_*j*_(*t*))^*T*^ *S*_*j*_(*s*)) for all *s*, *t* = 1,2,…, *m* and *j* = 1,2,…, *q*, we only need to prove that

(A.1) tr⁡((R(s))TR(t))=0, ∑j=1qtr⁡((Sj(s))TSj(t))=0,               1≤t<s≤m.

We prove the conclusion by induction, and two steps are required.

*Step  1*. we will show that

(A.2) tr⁡((R(k+1))TR(k))=0, ∑j=1qtr⁡((Sj(k+1))TSj(k))=0,                 k=1,2,…,m−1.

To prove this conclusion, we also use induction.

For *k* = 1, by Algorithm 3, we have that

(A.3) tr⁡((R(2))TR(1)) =tr⁡([R(1)−||R(1)||F2∑j=1q||Sj(1)||F2    ×diag⁡(∑j=1qA1jSj(1)B1j,∑j=1qA2jSj(1)B2j,…,        ∑j=1qApjSj(1)Bpj)]TR(1)) =||R(1)||F2−||R(1)||F2∑j=1q||Sj(1)||F2 ×tr⁡([diag⁡(∑j=1qA1jSj(1)B1j,        ∑j=1qA2jSj(1)B2j,…,∑j=1qApjSj(1)Bpj)]T    ·diag⁡(M1−∑l=1qA1lXl(1)B1l,       M2−∑l=1qA2lXl(1)B2l,…,       Mp−∑l=1qAplXl(1)Bpl))=||R(1)||F2−||R(1)||F2∑j=1q||Sj(1)||F2 ×tr⁡(diag⁡⁡((∑j=1qA1jSj(1)B1j)T         (M1−∑l=1qA1lXl(1)B1l),         (∑j=1qA2jSj(1)B2j)T         (M2−∑l=1qA2lXl(1)B2l),…,         (∑j=1qApjSj(1)Bpj)T         (Mp−∑l=1qAplXl(1)Bpl)))=||R(1)||F2−||R(1)||F2∑j=1q||Sj(1)||F2 ×tr⁡(∑i=1p((∑j=1qBijT(Sj(1))TAijT)      ×(Mi−∑l=1qAilXl(1)Bil)))=||R(1)||F2−||R(1)||F2∑j=1q||Sj(1)||F2 ×tr⁡(∑j=1q(Sj(1))T[∑i=1pAijT(Mi−∑l=1qAilXl(1)Bil)BijT])=||R(1)||F2−||R(1)||F2∑j=1q||Sj(1)||F2 ×tr⁡(∑j=1q(Sj(1))T    ×[∑i=1pAijT(Mi−∑l=1qAilXl(1)Bil)BijT2    +∑i=1pPjAijT(Mi−∑l=1qAilXl(1)Bil)BijTPj2])=||R(1)||F2−||R(1)||F2∑j=1q||Sj(1)||F2 ×tr⁡(∑j=1q(Sj(1))TSj(1))=0,∑j=1qtr⁡((Sj(2))TSj(1)) =∑j=1qtr⁡([12(∑i=1pAijT(Mi−∑l=1qAilXl(2)Bil)BijT       +∑i=1pPjAijT(Mi−∑l=1qAilXl(2)Bil)       ×BijTQj)      +||R(2)||F2||R(1)||F2Sj(1)]TSj(1))=∑j=1qtr⁡((∑i=1pAijT(Mi−∑l=1qAilXl(2)Bil)BijT     +||R(2)||F2||R(1)||F2Sj(1))TSj(1))=∑j=1qtr⁡((Sj(1))T∑i=1pAijT(Mi−∑l=1qAilXl(2)Bil)BijT) +||R(2)||F2||R(1)||F2∑j=1qtr⁡((Sj(1))TSj(1))=∑i=1ptr⁡(∑j=1q(Mi−∑l=1qAilXl(2)Bil)TAijSj(1)Bij) +||R(2)||F2||R(1)||F2∑j=1q||Sj(1)||F2=tr⁡(diag⁡((M1−∑l=1qA1lXl(2)B1l)T,      (M2−∑l=1qA2lXl(2)B2l)T,…,      (Mp−∑l=1qAplXl(2)Bpl)T)   ×diag⁡⁡(∑j=1qA1jSj(1)B1j,∑j=1qA2jSj(1)B2j,…,    ∑j=1qApjSj(1)Bpj))+||R(2)||F2||R(1)||F2∑j=1q||Sj(1)||F2=∑j=1q||Sj(1)||F2||R(1)||F2tr⁡((R(2))T(R(1)−R(2))) +||R(2)||F2||R(1)||F2∑j=1q||Sj(1)||F2=∑j=1q||Sj(1)||F2||R(1)||F2tr⁡((R(2))TR(1))=0.

Assume that (A.2) holds for *k* = *m* − 1; that is,

(A.4) tr⁡((R(m))TR(m−1))=0,∑j=1qtr⁡((Sj(m))TSj(m−1))=0.

When *k* = *m*, we have that

(A.5) tr⁡((R(m+1))TR(m)) =tr⁡([R(m)−||R(m)||F2∑j=1q||Sj(m)||F2    ×diag⁡(∑j=1qA1jSj(m)B1j,∑j=1qA2jSj(m)B2j,…,        ∑j=1qApjSj(m)Bpj)]TR(m))=||R(m)||F2−||R(m)||F2∑j=1q||Sj(m)||F2 ×tr⁡([diag⁡(∑j=1qA1jSj(m)B1j,∑j=1qA2jSj(m)B2j,…,        ∑j=1qApjSj(m)Bpj)]T    ·diag⁡(M1−∑l=1qA1lXl(m)B1l,       M2−∑l=1qA2lXl(m)B2l,…,       Mp−∑l=1qAplXl(m)Bpl))=||R(m)||F2−||R(m)||F2∑j=1q||Sj(m)||F2 ×tr⁡(diag⁡⁡((∑j=1qA1jSj(m)B1j)T         ×(M1−∑l=1qA1lXl(m)B1l),           (∑j=1qA2jSj(m)B2j)T          ×(M2−∑l=1qA2lXl(m)B2l),…,          (∑j=1qApjSj(m)Bpj)T          ×(Mp−∑l=1qAplXl(m)Bpl)))=||R(m)||F2−||R(m)||F2∑j=1q||Sj(m)||F2 ×tr⁡(∑i=1p((∑j=1qBijT(Sj(m))TAijT)      ×(Mi−∑l=1qAilXl(m)Bil)))=||R(m)||F2−||R(m)||F2∑j=1q||Sj(m)||F2 ×tr⁡(∑j=1q(Sj(m))T    ×[∑i=1pAijT(Mi−∑l=1qAilXl(m)Bil)BijT])=||R(m)||F2−||R(m)||F2∑j=1q||Sj(m)||F2 ×tr⁡(∑j=1q(Sj(m))T     ×[∑i=1pAijT(Mi−∑l=1qAilXl(m)Bil)BijT2      +∑i=1pPjAijT(Mi−∑l=1qAilXl(m)Bil)BijTPj2])=||R(m)||F2−||R(m)||F2∑j=1q||Sj(m)||F2 ×tr⁡(∑j=1q(Sj(m))T(Sj(m)−||R(m)||F2||R(m−1)||F2Sj(m−1)))=0,

(A.6) ∑j=1qtr⁡((Sj(m+1))TSj(m)) =∑j=1qtr⁡([12(∑i=1pAijT(Mi−∑l=1qAilXl(m+1)Bil)BijT       +∑i=1pPjAijT(Mi−∑l=1qAilXl(m+1)Bil)       ×BijTPj)      +||R(m+1)||F2||R(m)||F2Sj(m)]TSj(m)) =∑j=1qtr⁡((∑i=1pAijT(Mi−∑l=1qAilXl(m+1)Bil)BijT      +||R(m+1)||F2||R(m)||F2Sj(m))TSj(m)) =∑j=1qtr⁡((Sj(m))T∑i=1pAijT(Mi−∑l=1qAilXl(m+1)Bil)BijT)  +||R(m+1)||F2||R(m)||F2∑j=1qtr⁡((Sj(m))TSj(m)) =∑i=1ptr⁡(∑j=1q(Mi−∑l=1qAilXl(m+1)Bil)TAijSj(m)Bij)  +||R(m+1)||F2||R(m)||F2∑j=1q||Sj(m)||F2 =tr⁡(diag⁡((M1−∑l=1qA1lXl(m+1)B1l)T,       (M2−∑l=1qA2lXl(m+1)B2l)T,…,       (Mp−∑l=1qAplXl(m+1)Bpl)T)   ×diag⁡⁡(∑j=1qA1jSj(m)B1j,∑j=1qA2jSj(m)B2j,…,    ∑j=1qApjSj(m)Bpj))  +||R(m+1)||F2||R(m)||F2∑j=1q||Sj(m)||F2 =∑j=1q||Sj(m)||F2||R(m)||F2  ×tr⁡((R(m+1))T(R(m)−R(m+1)))  +||R(m+1)||F2||R(m)||F2∑j=1q||Sj(m)||F2 =∑j=1q||Sj(m)||F2||R(m)||F2tr⁡((R(m+1))TR(m))=0.

Hence, (A.2) holds for *k* = *m*. Therefore, (A.2) holds by the principle of induction.

*Step  2.* We show that

(A.7) tr⁡((R(k+1))TR(t))=0,∑j=1qtr⁡((Sj(k+1))TSj(t))=0,   t=1,2,… ,k,  ∀k≥1.

When *k* = 1, (A.7) holds.

Assume that

(A.8) tr⁡((R(k))TR(t))=0,  ∑j=1qtr⁡((Sj(k))TSj(t))=0,           t=1,2,…,k−1,  ∀k≥2;

then we show that

(A.9) tr⁡((R(k+1))TR(t))=0,∑j=1qtr⁡((Sj(k+1))TSj(t))=0,      t=1,2,…,k.

In fact, we have that

(A.10) tr⁡((R(k+1))TR(t)) =tr⁡([R(k)−||R(k)||F2∑j=1q||Sj(k)||F2    ×diag⁡(∑j=1qA1jSj(k)B1j,∑j=1qA2jSj(k)B2j,…,        ∑j=1qApjSj(k)Bpj)]TR(t))   =tr⁡((R(k))TR(t))−||R(k)||F2∑j=1q||Sj(k)||F2  ×tr⁡([diag⁡(∑j=1qA1jSj(k)B1j,         ∑j=1qA2jSj(k)B2j,…,∑j=1qApjSj(k)Bpj)]T    ·diag⁡(M1−∑l=1qA1lXl(t)B1l,       M2−∑l=1qA2lXl(t)B2l,…,       Mp−∑l=1qAplXl(t)Bpl)) =−||R(k)||F2∑j=1q||Sj(k)||F2   ×tr⁡(diag⁡((∑j=1qA1jSj(k)B1j)T      ×(M1−∑l=1qA1lXl(t)B1l),       (∑j=1qA2jSj(k)B2j)T      ×(M2−∑l=1qA2lXl(t)B2l),…,      (∑j=1qApjSj(k)Bpj)T      ×(Mp−∑l=1qAplXl(t)Bpl))) =−||R(k)||F2∑j=1q||Sj(k)||F2   ×tr⁡(∑i=1p((∑j=1qBijT(Sj(k))TAijT)        ×(Mi−∑l=1qAilXl(t)Bil))) =−||R(k)||F2∑j=1q||Sj(k)||F2   ×tr⁡(∑j=1q(Sj(k))T[∑i=1pAijT(Mi−∑l=1qAilXl(t)Bil)BijT]) =−||R(k)||F2∑j=1q||Sj(k)||F2   ×tr⁡(∑j=1q(Sj(k))T     ×[∑i=1pAijT(Mi−∑l=1qAilXl(t)Bil)BijT2     +∑i=1pPjAijT(Mi−∑l=1qAilXl(t)Bil)BijTPj2]) =−||R(k)||F2∑j=1q||Sj(k)||F2   ×tr⁡(∑j=1q(Sj(k))T(Sj(t)−||R(t)||F2||R(t−1)||F2Sj(t−1))) =−||R(k)||F2∑j=1q||Sj(k)||F2∑j=1qtr⁡((Sj(k))TSj(t))  +||R(k)||F2∑j=1q||Sj(k)||F2||R(t)||F2||R(t−1)||F2  ×∑j=1qtr⁡((Sj(k))TSj(t−1))=0.

From the above results, we have tr⁡(*R*(*k* + 1)^*T*^ *R*(*t* + 1)) = 0, *t* = 1,2,…, *k* − 1, and

(A.11) ∑j=1qtr⁡((Sj(k+1))TSj(t)) =∑j=1qtr⁡([12(∑i=1pAijT(Mi−∑l=1qAilXl(k+1)Bil)BijT     +∑i=1pPjAijT(Mi−∑l=1qAilXl(k+1)Bil)BijTPj)     +||R(k+1)||F2||R(k)||F2Sj(k)]TSj(t)) =∑j=1qtr⁡((∑i=1pAijT(Mi−∑l=1qAilXl(k+1)Bil)BijT      +||R(k+1)||F2||R(k)||F2Sj(k))TSj(t)) =∑j=1qtr⁡((Sj(t))T∑i=1pAijT(Mi−∑l=1qAilXl(k+1)Bil)BijT)  +||R(k+1)||F2||R(k)||F2∑j=1qtr⁡((Sj(k))TSj(t)) =∑i=1ptr⁡(∑j=1q(Mi−∑l=1qAilXl(k+1)Bil)TAijSj(t)Bij) =tr⁡(diag⁡((M1−∑l=1qA1lXl(k+1)B1l)T,      (M2−∑l=1qA2lXl(k+1)B2l)T,…,      (Mp−∑l=1qAplXl(k+1)Bpl)T)    ×diag⁡(∑j=1qA1jSj(t)B1j,       ∑j=1qA2jSj(t)B2j,…,∑j=1qApjSj(t)Bpj)) =∑j=1q||Sj(t)||F2||R(t)||F2  ×tr⁡((R(k+1))T(R(t)−R(t+1))) =∑j=1q||Sj(t)||F2||R(t)||F2  ×tr⁡((R(k+1))TR(t))=0.

By the principle of induction, (A.7) holds.

Note that (A.1) is implied in Steps 1 and 2 by the principle of induction. This completes the proof.

### B. The Proof of Lemma 5

We prove the conclusion by induction for the positive integer *k*.

For *k* = 1, we have that

(B.1) ∑j=1qtr⁡((Xj∗−Xj(1))TSj(1)) =∑j=1qtr⁡((Xj∗−Xj(1))T     ×[12(∑i=1pAijT(Mi−∑l=1qAilXl(1)Bil)BijT       +∑i=1pPjAijT(Mi−∑l=1qAilXl(1)Bil)BijTPj)]) =∑j=1qtr⁡((Xj∗−Xj(1))T     ×[∑i=1pAijT(Mi−∑l=1qAilXl(1)Bil)BijT]) =∑i=1ptr⁡((Mi−∑l=1qAilXl(1)Bil)T     ×∑j=1qAij(Xj∗−Xj(1))Bij) =tr⁡(diag⁡((M1−∑l=1qA1lXl(1)B1l)T,       (M2−∑l=1qA2lXl(1)B2l)T,…,       (Mp−∑l=1qAplXl(1)Bpl)T)    ×diag⁡(∑j=1qA1j(Xj∗−Xj(1))B1j,       ∑j=1qA2j(Xj∗−Xj(1))B2j,…,       ∑j=1qApj(Xj∗−Xj(1))Bpj)) =tr⁡(diag⁡((M1−∑l=1qA1lXl(1)B1l)T,      (M2−∑l=1qA2lXl(1)B2l)T,…,      (Mp−∑l=1qAplXl(1)Bpl)T)     ×diag⁡⁡(M1−∑j=1qA1jXj(1)B1j,         M2−∑j=1qA2jXj(1)B2j,…,         Mp−∑j=1qApjXj(1)Bpj)) =||R(1)||2.

Assume that (9) holds for *k* = *m*. When *k* = *m* + 1, by Algorithm 3, we have that

(B.2) ∑j=1qtr⁡((Xj∗−Xj(m+1))TSj(m+1))=∑j=1qtr⁡((Xj∗−Xj(m+1))T    ×[12(∑i=1pAijT(Mi−∑l=1qAilXl(m+1)Bil)BijT      +∑i=1pPjAijT(Mi−∑l=1qAilXl(m+1)Bil)BijTPj)      +||R(m+1)||F2||R(m)||F2Sj(m)])=∑j=1qtr⁡((Xj∗−Xj(m+1))T    ×[∑i=1pAijT(Mi−∑l=1qAilXl(m+1)Bil)BijT]) +||R(m+1)||F2||R(m)||F2 ×∑j=1qtr⁡((Xj∗−Xj(m+1))TSj(m))=∑i=1ptr⁡((Mi−∑l=1qAilXl(m+1)Bil)T    ×∑j=1qAij(Xj∗−Xj(m+1))Bij) +||R(m+1)||F2||R(m)||F2∑j=1qtr⁡((Xj∗−Xj(m))TSj(m)) −||R(m+1)||F2∑j=1q||Sj(m)||F2∑j=1qtr⁡((Sj(m))TSj(m))=tr⁡(diag⁡((M1−∑l=1qA1lXl(m+1)B1l)T,     (M2−∑l=1qA2lXl(m+1)B2l)T,…,     (Mp−∑l=1qAplXl(m+1)Bpl)T)   ×diag⁡⁡(∑j=1qA1j(Xj∗−Xj(m+1))B1j,       ∑j=1qA2j(Xj∗−Xj(m+1))B2j,…,       ∑j=1qApj(Xj∗−Xj(m+1))Bpj)) +||R(m+1)||F2||R(m)||F2||R(m)||F2 −||R(m+1)||F2∑j=1q||Sj(m)||F2∑j=1q||Sj(m)||F2=tr⁡(diag⁡((M1−∑l=1qA1lXl(m+1)B1l)T,     (M2−∑l=1qA2lXl(m+1)B2l)T,…,     (Mp−∑l=1qAplXl(m+1)Bpl)T)   ×diag⁡⁡(M1−∑j=1qA1jXj(m+1)B1j,       M2−∑j=1qA2jXj(m+1)B2j,…,       Mp−∑j=1qApjXj(m+1)Bpj)) +||R(m+1)||F2−||R(m+1)||F2=||R(m+1)||F2.

Therefore, (9) holds for *k* = *m* + 1. Thus (9) holds by the principal of induction. This completes the proof.

## References

[1] Ding F, Chen T (2006). On iterative solutions of general coupled matrix equations. *SIAM Journal on Control and Optimization*.

[2] Ding F, Chen T (2005). Gradient based iterative algorithms for solving a class of matrix equations. *IEEE Transactions on Automatic Control*.

[3] Ding F, Liu PX, Ding J (2008). Iterative solutions of the generalized Sylvester matrix equations by using the hierarchical identification principle. *Applied Mathematics and Computation*.

[4] Ding J, Liu Y, Ding F (2010). Iterative solutions to matrix equations of the form *A*
_*i*_ × *B*
_*i*_ = *F*
_*i*_. *Computers and Mathematics with Applications*.

[5] Xie L, Ding J, Ding F (2009). Gradient based iterative solutions for general linear matrix equations. *Computers and Mathematics with Applications*.

[6] Ding F, Chen T (2005). Iterative least-squares solutions of coupled Sylvester matrix equations. *Systems and Control Letters*.

[7] Wu AG, Feng G, Duan GR, Wu WJ (2010). Iterative solutions to coupled Sylvester-conjugate matrix equations. *Computers and Mathematics with Applications*.

[8] Wu AG, Li B, Zhang Y, Duan GR (2011). Finite iterative solutions to coupled Sylvester-conjugate matrix equations. *Applied Mathematical Modelling*.

[9] Wu AG, Feng G, Duan GR, Wu WJ (2010). Finite iterative solutions to a class of complex matrix equations with conjugate and transpose of the unknowns. *Mathematical and Computer Modelling*.

[10] Jonsson I, Kågström B (2002). Recursive blocked algorithms for solving triangular systems—part I: one-sided and coupled Sylvester-type matrix equations. *ACM Transactions on Mathematical Software*.

[11] Jonsson I, Kågström B (2002). Recursive blocked algorithms for solving triangular systems—part II: two-sided and generalized Sylvester and Lyapunov matrix equations. *ACM Transactions on Mathematical Software*.

[12] Dehghan M, Hajarian M (2010). The general coupled matrix equations over generalized bisymmetric matrices. *Linear Algebra and Its Applications*.

[13] Huang GX, Wu N, Yin F, Zhou ZL, Guo K (2012). Finite iterative algorithms for solving generalized coupled Sylvester systems—part I: one-sided and generalized coupled Sylvester matrix equations over generalized reflexive solutions. *Applied Mathematical Modelling*.

[14] Yin F, Huang GX, Chen DQ (2012). Finite iterative algorithms for solving generalized coupled Sylvester systems—part II: two-sided and generalized coupled Sylvester matrix equations over reflexive solutions. *Applied Mathematical Modelling*.

[15] Cai J, Chen GX (2009). An iterative algorithm for the least squares bisymmetric solutions of the matrix equations *A*
_1_ × *B*
_1_ = *C*
_1_, *A*
_2_ × *B*
_2_ = *C*
_2_. *Mathematical and Computer Modelling*.

[16] Chen D, Yin F, Huang G (2012). An iterative algorithm for the generalized reflexive solution of the matrix equations *A* × *B* = *E*, *C* × *D* = *F*. *Journal of Applied Mathematics*.

[17] Ding F, Chen T (2005). Hierarchical gradient-based identification of multivariable discrete-time systems. *Automatica*.

[18] Ding F, Chen T (2005). Hierarchical least squares identification methods for multivariable systems. *IEEE Transactions on Automatic Control*.

[19] Ding F, Chen T (2005). Hierarchical identification of lifted state-space models for general dual-rate systems. *IEEE Transactions on Circuits and Systems I*.

[20] Huang GX, Yin F, Guo K (2008). An iterative method for the skew-symmetric solution and the optimal approximate solution of the matrix equation *A* × *B* = *C*. *Journal of Computational and Applied Mathematics*.

[21] Liao AP, Lei Y (2005). Least-squares solution with the minimum-norm for the matrix equation (*A* × *B*
, *G* × *H*
) = (C, *D*). *Computers and Mathematics with Applications*.

[22] Peng ZH, Hu XY, Zhang L (2006). An efficient algorithm for the least-squares reflexive solution of the matrix equation *A*
_1_ × *B*
_1_ = *C*
_1_, *A*
_2_ × *B*
_2_ = *C*
_2_. *Applied Mathematics and Computation*.

[23] Wang QW (2005). Bisymmetric and centrosymmetric solutions to systems of real quaternion matrix equations. *Computers and Mathematics with Applications*.

[24] Wang QW (2005). The general solution to a system of real quaternion matrix equations. *Computers and Mathematics with Applications*.

[25] Wang QW, Li CK (2009). Ranks and the least-norm of the general solution to a system of quaternion matrix equations. *Linear Algebra and Its Applications*.

[26] Yin F, Huang GX (2012). An iterative algorithm for the least squares generalized reflexive solutions of the matrix equations *A* × *B* = *E*, *C* × *D* = *F*. *Abstract and Applied Analysis*.

[27] Yin F, Huang G (2012). An iterative algorithm for the generalized reflexive solutions ofthe generalized coupled Sylvester matrix equations. *Journal of Applied Mathematics*.

[28] Zhou B, Li ZY, Duan GR, Wang Y (2009). Weighted least squares solutions to general coupled Sylvester matrix equations. *Journal of Computational and Applied Mathematics*.

